# Self-care practice and associated factors among adult asthmatic patients on follow-up care at Northwest Amhara referral hospitals, Northwest Ethiopia 2020

**DOI:** 10.1186/s12890-021-01508-4

**Published:** 2021-04-29

**Authors:** Sosna Melkamu Abegaz, Mulugeta Wassie, Abere Woretaw Azagew

**Affiliations:** 1 Medical Nursing, University of Gondar, Gondar, Ethiopia; 2 Department of Medical Nursing, School of Nursing, College of Medicine and Health Sciences, University of Gondar, Gondar, Ethiopia

**Keywords:** Asthma, Self-care practice, Northwest Amhara referral hospitals, Ethiopia

## Abstract

**Background:**

Self-care practice of asthma is the strategy for asthma symptom control and future reduction of exacerbation, but it is poorly implemented in clinical settings due to the patients, professionals, and organizational related factors. Therefore, the study aimed to assess the self-care practice and associated factors among adult asthmatic patients at Northwest Amhara referral hospitals.

**Methods:**

Institution-based cross-sectional study was conducted among asthmatic patients on follow-up care at Northwest Amhara Regional State referral hospitals from February 1st, 2020 to March 30, 2020. Data were collected through an interviewer-administered technique. Asthma self-care practice tool was used to measure the outcome. Data were entered into EPI info version 7 and exported to SPSS version 22 for analysis. A binary logistic regression analysis was used. In multivariable logistic regression analysis, those independent variables having *p* value < 0.05 were considered as statistically significant with poor self-care practice of asthma.

**Results:**

A total of 470 participants enrolled in the study with a response rate of 100%. The proportion of good self-care practice among asthmatic patients was found to be 42.3%. The study revealed that; age group ≥ 55 years, having a co-morbid illness and borderline anxiety, having no social support, and drinking alcohol were significantly associated with poor asthma self-care practice.

**Conclusions:**

Poor-self care practice in this study was high. Efforts need to be implemented for asthmatic patients with older age, having co-morbid illness and borderline anxiety, having no social support, and drinking alcohol.

## Background

Asthma is a heterogeneous disease usually characterized by chronic airway inflammation. It is one of the commonest non-communicable diseases. Globally, there are around 235 million people affected by asthma [1, 2], and 0.18 million people death recorded annually [3]. In Africa, the burden of asthma increased due to the fastest rate of urbanizations [4, 5]. In Africa like Cameron, Uganda, and Congo are appearing to have high asthma with a prevalence of 2.7%, 11.0%, and 6.9% respectively [6–8]. Studies carried out in Jimma, Debrebrhan, and Addis Ababa, Ethiopia indicated that the prevalence of asthma was 4.9%, 29.6%, and 1.04% respectively, due to geographical variations [9–11]. Symptom control and reduction of future risk of asthma exacerbation are the domains of asthma treatment strategies. The self-care practice of asthma is an established effective and guideline-recommended approach to control exacerbation [12]. It may relieve the symptom of asthma and permits the patient to carry out the promotion of health, cope with illness, and normal social life [13]. Asthma self-care practice remains poorly implemented in clinical settings due to patients, professionals, and organizational related factors despite evidence of improved healthcare outcomes [14]. Poorly controlled asthma resulted in a considerable burden and is a serious public health problem in the developing world [15]. The expense of uncontrolled asthma is more than twice as high as that of a patient with controlled asthma [16].

According to the 2016 Global Initiative for Asthma report, lack of patients’ adherence to the recommended treatment strategy is associated with uncontrolled asthma. Uncontrolled asthma is burdensome to patients and at risk for future asthma exacerbation. Asthma treatment requires a partnership between the person with asthma and their health care providers [17], but its treatment outcome depends on the presence of co-morbid illness, exposure to aggravations, and adherence to the prescribed treatment [18, 19].

In Ethiopia, the prevalence of controlled asthma was low which ranged from 43.8 to 76.1% [20–22]. Psychological conditions such as anxiety and depression occur more commonly in people with poorly controlled asthma [23]. Poorly controlled asthma has an impact on outdoor daily activity in terms of limitations [24], decreased social interaction, negative emotion, and worsening of asthma symptoms [25]. Alongside, it has frequent unplanned visits to the emergency department, long-time hospitalizations, and reduction in quality of life [26].

Despite there are the availability of self-care management recommendations, but its implementation of self care-practice was not well studied. Therefore, the study aimed to assess self-care practice and associated factors among asthmatic patients on follow-up care in Northwest Amhara Regional State referral hospitals, Northwest Ethiopia.

## Methods

### Study design, study setting, and period

An institutional-based cross-sectional study was used. The study was conducted among asthmatic patients who attended Northwest Amhara Regional State referral hospitals from February 1st/2020 to March 30, 2020. There were around three referral hospitals found in Amhara Regional State namely; University of Gondar Specialized Hospital (UoGSH), Felege Hiwot Referral Hospital (FHRH), and Debre Markos Referral Hospital (DMRH).

Each of them serves about 5–7 million people in their catchment area. The University of Gondar Specialized Hospital is found in the Northern part of Amhara Region in Gondar town, FHCRH is found in Bahir Dar city which is the capital city of Amhara Regional state, and DMRH is found in East of Gojam of Amhara Regional State, Northwest Ethiopia. In those hospitals there are around 470 asthmatic patients get follow-up care service of which 150 asthmatic patients were at UoGSH, 120 were at DMRH, and 200 were at FHRH.

### Source and study population

Asthmatic patients whose age greater than or equal to 18 years old and who has been on follow-up care at Northwest Amhara Region referral hospital were taken as the source population and patients who had follow-up care in the selected hospitals during the data collection period were considered as the study population.

### Sample size and sampling procedure

The sample size was determined using the single population proportion formula through the Epi Info Stat Calc program with the assumption of; 95% level of confidence, 5% margin of error, and 50% of the proportion of Asthma self-care practice. Taking these assumptions, the estimated sample size was 384. Considering a 10% non-response rate, the final sample size was 423. Since the determined sample size was approximately equal to the source population, all 470 asthmatic patients on follow-up care were included in the study using the census sampling method.

### Data collection tools and procedures

Data were collected by the interviewer-administer technique using validated tools drived from previous literature [27–31]. The tools cover the following aspects: socio-demographic factors, asthmatic self-care practice assessment, asthmatic medication assessment, behavioral related factors, social support, anxiety and depression, and asthma knowledge. The authors used an Asthmatic self-care practice questionnaire to measure the outcome [28]. The tool is a validated tool and the pretest was conducted to evaluate the clarity of the tool. It has eight items. For each item, participants rated their self-care practice on a four-point scale ranging from one (never perform) to four (always perform). The total score ranges from eight to thirty-two and is calculated by summing the scores for each item. Participants who scored above or equal to the mean were considered as good asthma self-care practice whereas below the mean were taken as a poor asthma self-care practice.

Medication adherence was measured by the Medication Adherence Report Scale for Asthma (MARS-A). This contains ten items and for each item, patients rated their self-care practice on a 5-point scale ranging from one (always) to five (never). The total score ranges from 10 to 50 and is calculated by summing the scores for each item. Medication adherence was measured as participants who scored with the MARS-A above or equal to the mean was taken as having good adherence and those who scored below the mean considered as having poor adherence [29].

The behavioral status of the participants was assessed using a history of cigarette smoking and alcohol drinking habits. Social support was measured by using the Multidimensional Scale of Perceived Social Support [31]. The multidimensional scale of the perceived social support tool consisted of twelve items. For each item, participants rated on a seven-point scale from one (strongly disagree) to seven (strongly agree). The total score ranges from twelve to eighty four which was calculated by summing up the scores for each item. Participants who scored above or equal to the mean from multidimensional social support questions were referred to as having social support and those who scored below the mean considered as having no social support.

Anxiety and depression were measured by using the Hospital Anxiety and Depression Scale (HADS) [29]. The HADS consisted of fourteen items. Participants who scored between 0–7, 8–10, and 11–21 were taken as having normal, borderline, and higher levels of anxiety and depression respectively.

The knowledge of asthma self-care practice was measured by using the Knowledge of Asthma Self-care Questionnaire (KASQ > 50) [27]. Participants who scored greater than or equal to the mean of knowledge-related questions were categorized as having good knowledge and those who scored below the mean were considered as having poor knowledge. Five nurses (three data collectors and two supervisors) participated in this research.

### Data quality measures

To ensure the quality of data, pre-test was conducted before the actual date of data collection period. The questionnaire was prepared first in English and translated into Amharic (local language) for data collection and then back to English for analysis to maintain its consistency. The training was given to data collectors and supervisors.

### Data processing and analysis

Data were checked, coded, and entered into Epi Info version seven, and exported to SPSS version twenty for analysis. Descriptive statistics such as mean, median, interquartile range (IQR), frequency, and percentage were used. A binary logistic regression analysis was used to identify factors associated with the outcome variable. All independent variables were entered into multivariable logistic regression analysis. Variables having a *p* value < 0.05 in the multivariable logistic regression analysis were considered statistically significant. Hosmer’s and Lemishow goodness of fit test was used to test the model fitness.

## Results

### Socio-demographic characteristics of the study participants

A total of 470 participants were enrolled in the study, making a 100% response rate. The median (IQR) age of participants was 47 (IQR 35–58) years. Two hundred thirteen (45.3%) of the study participants were in the age range of 35–54 years. Among study participants; 260 (55.3%) were females, more than half (58.9%) were married, 369(78.5) were Orthodox Christian by religion, 453(96.4%) were Amhara by ethnicity, 170(36.2%) attended college and above education level, 356 (75.7%) were urban dwellers, and 110 (23.4%) were currently civil servants. The median average monthly household income was 77 USD (IQR 35.26–78.2USD) (Table 1).

**Table 1:** Socio-demographic characteristics of adult asthmatic patients at Amhara Regional State referral hospitals follow-up care clinic, Northwest Ethiopia, 2020 (n = 470)

Variables	Frequency (n)	Percent (%)
Sex		
Male	210	44.7
Female	260	55.3
Age groups(years)		
18–34	109	23.2
35–54	213	45.3
> 55	148	31.5
Marital status		
Single	102	21.7
Married	277	58.9
Divorced	31	6.6
Widowed	60	12.8
Religion		
Orthodox	369	78.5
Muslim	62	13.2
Protestant	39	8.3
Ethnicity		
Amhara	453	96.4
Oromo	5	1.1
Tigray	12	2.6
Education status		
Unable to read and write	142	30.2
Primary school	77	16.4
Secondary school	81	17.2
College and above	170	36.2
Residence		
Urban	356	75.5
Rural	114	24.3
Occupational status		
Student	42	8.9
Daily labourer	43	9.1
Farmer	57	12.1
Merchant	96	20.4
Housewife	97	20.6
Civil servant	110	23.4
	25	5.3
Average monthly income(USD)		
≤ 50	57	12.1
51–60	88	18.7
61–70	106	22.6
71–80	120	25.5
81–90	49	10.4
90–100	43	9.1
> 101	7	1.5

*USD* United States Dollar

### Clinical characteristic of the study participants

Regarding the respondent’s clinical characteristics, more than half of the study participants (56.6%) had diagnosed asthma at the age range of 25–49 years. One hundred seventy-nine (38.1%) of them had 11–20 years duration of asthma, 200(42.6%) had a family history of asthma. Seventy-seven (16.4%) of the study participants had co-morbid illnesses of which 36(7.7%) had chronic heart failure. The majority (88.9%) of the participants had a history of frequent asthma exacerbation with ≥ 2exacerbations per year. Three hundred sixty-eight (78.3%) of the study participants adhered to the prescribed asthmatic medications (Table 2).

**Table 2:** Clinical characteristics of adult asthmatic patients at Amhara Regional State referral hospitals follow-up care clinic, Northwest Ethiopia, 2020 (n = 470)

Variables	Frequency (n)	Percent (%)
Age at asthma diagnosis (year)		
< 25	145	30.9
25–49	266	56.6
> 50	59	12.6
Duration living with asthma (year)		
< 2	21	4.5
2–5	51	10.9
6–10	110	23.4
11–20	179	38.1
> 20	107	23.2
Family history of asthma		
Yes	200	42.6
No	270	57.4
History of co-morbidity		
Yes	393	83.6
No	77	16.4
Types of co-morbidity		
Chronic heart failure	36	7.7
Diabetes mellitus	10	2.1
Chronic liver disease	5	1.1
Chronic renal disease	2	0.4
Hypertension	21	4.5
HIV/AIDS	3	0.6
History of asthma exacerbation		
Yes	418	88.9
No	52	11.1
Adherence to asthmatic medications		
Adhered	368	78.3
Not adhered	102	21.7

*HIV/AIDS* human immunodeficiency virus/acquired immune deficiency syndrome

### Knowledge, psychological, and behavioral characteristics of the study participants

Among the study participants; 275(58.5%) were not knowledgeable about asthma self-care practice and nearly 7% and 11.3% of study participants had anxiety and depression respectively.

Above three-fourths (75.5%) of participants were ever drunk alcohol, 20(4.3%) smoked a cigarette, and 389(82.8%) had not physical exercise habit (Table 3).

**Table 3:** Knowledge, psychological and behavioral characteristics of adult asthmatic patients at Amhara Regional State referral hospitals follow-up care clinic, Northwest Ethiopia, 2020 (n = 470)

Variables	Frequency(n)	Percent (%)
Knowledge		
Knowledgeable	195	41.5
Not knowledgeable	275	58.5
Anxiety		
Normal	229	48.7
Borderline	209	44.5
Abnormal	32	6.8
Depression		
Normal	311	66.2
Borderline	106	22.6
Abnormal	53	11.3
Social support		
Supported	337	71.7
Not supported	133	28.3
History of drinking alcohol		
Yes	355	75.5
No	115	24.5
Current drinking of alcohol		
Yes	101	21.5
No	369	78.5
Types of alcohol used		
Local Tela	48	10.2
Tej	13	2.7
Areki	16	3.4
National beer	24	5.1
Alcohol drinking per week		
> 2 times	22	4.7
≤ 2 times	79	16.8
Currently smoked		
Yes	20	4.3
No	450	95.7
No. of cigarettes smoked per day		
< 5	11	2.3
5–6	3	0.6
> 11	6	1.3
Exercise habit		
Yes	81	17.2
No	389	82.8

### The proportion of self-care practice

**Fig. 1:**
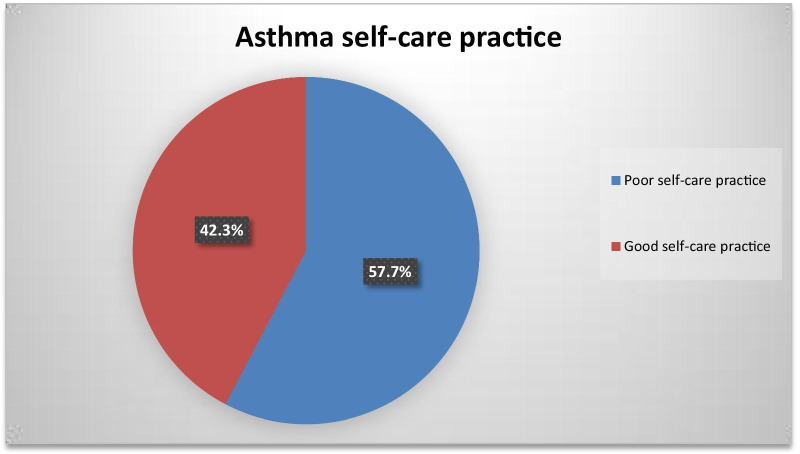
The proportion of good self-care practice status among adults asthmatic patients attending at Amhara Regional State referral hospitals follow-up care clinic, Northwest Ethiopia, 2020 (n = 470)

The proportion of good self-care practice among asthmatic patients was found to be 42.3%(37.9, 46.6%) (Fig. 1).

### Factors associated with self-care practice

Regarding the factors; the age group ≥ 55 years (AOR = 2.463, 95%, CI 1.339–4.334, having a co-morbid illness (AOR = 1.860, 95% CI 1.063–3.254), having borderline anxiety (AOR = 1.740, 95% CI 1.144–2.645), having social support (AOR = 1.915, 95% CI 1.193–3.254) and having a history of alcohol drank (AOR = 1.832, 95% CI 1.136–2.954) were statistically associated with poor self-care practice (Table 4).

**Table 4:** Bivariate and multivariate logistic regression analysis for factors associated with poor self-care practice among patients with asthmatic at follow-up clinics in Northwest Ethiopia 2020 (n = 470)

Variables	Self Care Practice		COR 95% CI	AOR 95% CI
	Poor	Good		
Sex				
Male	129	81	1	
Female	142	118	0.756(0.552–1.094)	
Age groups (year)				
18–34	54	55	1	1
35–54	122	91	1.365(0.859–2.170)	1.288(0.785–2.113)
> 55	95	53	1.826(1.103–3.022)	2.463(1.339–4.334)*
Educational status				
Unable to read and write	74	68	0.708(0.451–1.111)	
Primary school	43	34	0.823(0.477–1.419)	
Secondary school	51	30	1.106(0.641–1.909)	
College and above	103	67	1	
Social support				
Not supported	89	44	1.723(1.132–2.622)	1.915(1.193–3.254)*
Supported	182	155	1	1
Anxiety				
Normal	117	122	1	1
Borderline	142	67	2.029(1.375–2.994)	1.740(1.144–2.645)*
Abnormal	12	20	0.574(0.268–1.230)	0.454(0.204–1.014)
Depression				
Normal	186	125	1	
Borderline	58	48	0.812(0.521–1.267)	
Abnormal	27	26	0.698(0.389–1.252)	
Knowledge				
Not knowlegeable	150	125	0.734(0.505–1.067)	
Knowlegeable	121	74	1	
Co-morbid illness				
No	232	161	1	1
Yes	39	38	1.404(0.860–2.292)	1.860(1.063–3.254)*
Duration of asthma (year)				
> 2	10	11	0.527(0.206–1.350)	
2–5	29	22	0.764(0.388–1.504)	
6–10	59	51	0.674(0.391–1.151)	
11–20	104	75	0.804(0.493–1.312)	
> 20	69	40	1	
Alcohol drinking				
No	221	148	1	1
Yes	50	51	1.523(0.979–2.370)	1.832(1.136–2.954)*
Physical exercise habit				
No	219	170	0.718(0.437–1.180)	
Yes	52	29	1	

*AOR* adjusted odds ratio, *CI* confidence interval, *COR* crude odds ratio

*p value < 0.05

## Discussion

The proportion of good self-care practice among asthmatic patients was found to be 42.3%(37.9, 46.6%). The finding of this study is in line with a study conducted in Congo 44% [32]. On the other hand, it was lower than the study conducted in Bangladesh 49.63% [27], Saudi Arabia 57.1% [28], and Taiwan 51.5% [33]. The possible reasons for the difference might be due to the study setting, sample size, sampling techniques, study period, the study population of the study includes age 18 years and below, socio-cultural variation, and lifestyle. On the contrary, the finding of this study was higher than the study conducted in India 34% [34]. The poor self-care practice may be related to poor health care attention, forgetfulness to medication intake, and limitation to health care resources.

Older age is the predictor of self-care practice for asthmatic patients. Asthmatic patients whose age ≥ 55 years were 2.463 times more likely to have poor self-care practice compared to asthmatic patients whose age group 18–34 years. The result was supported by a study in Taiwan [33]. Aging may result in molecular and cellular damage over time which leads to a gradual decrease in physical and mental capacity on the risk of disease. Older asthmatic patients may experience forgetfulness and decrease attention spam towards their health care. Aging-related problems such as heart diseases and obesity in the elderly increased the frequency as well as the severity of asthma exacerbation. There are also age-related issues leading to decrease control such as non-adherence, difficulty to use inhalers, and corticosteroids related side effects which hinder asthma controls in higher age groups [35]

Asthmatic patients who had anxiety were 1.74 times more likely to have poor self-care practice compared to those asthmatic patients who had no anxiety. This result was supported by a study conducted in Italy [36]. Anxiety might lead to neglect and impair recognition to do self-care. Anxiety involves an ongoing disproportionately strong feeling of fear, worry, or stress which can happen in response to chronic illness [37].

Asthmatic patients who had no social support were nearly two times more likely to have poor self-care practice compared to those who had social support. Social support from families and friends is important for persons with chronic illness such as asthmatics to develop a positive self-concept and self-esteem to give emotional and social support for the implementation of self-care practice. Social support improves the extent of self-management of asthma particularly in medication adherence [38].

Asthmatic patients who had a co-morbid illness were nearly two times more likely to have poor self-care practices compared to those patients who have no comorbid illness. Co-morbidities can worsen the conditions of the patient and make them unable to adhere to self-care activities. It can complicate the diagnosis and the management of asthma. The symptom of co-morbid conditions may be similar to those associated with poor asthma control which can lead to misdiagnosis and under treatment or over treatment [39].

The asthmatic patients who drink alcohol were nearly two times more likely to develop poor self-care practices compared to those asthmatic patients who didn’t drink alcohol. Drinking alcohol particularly wine appeared to be an important trigger for asthmatic response to be worsened. It also causes alcohol-induced asthma. Furthermore, alcohol intake can lead to pathological bronchoconstriction that affects many people with asthma [40]. As a result, asthmatic patients who drank alcohol might have poor self-care practice.

## Conclusions

The proportion of poor self-care practice in this study was high. Efforts need to be implemented for asthmatic patients with older age, having; comorbid illness, borderline anxiety, no social support, and a history of alcohol drinking.

## Data Availability

The datasets used and/or analysed during the current study are available from the corresponding author on reasonable request.

## Abbreviations

AIDS: Acquired immune deficiency syndrome
AOR: Adjusted odds ratio
CI: Confidence interval
COR: Crude odds ratio
DMRH: Debre-Markos referral hospital
FHRH: Felege Hiwot referral hospital
HADS: Hospital Anxiety and Depression Scale
HIV: Human immunodeficiency virus
UoGSH: University of Gondar Specialized Hospital
